# Quarterly Periscope

**Published:** 1823-09-01

**Authors:** 


### Quarterly Periscope

OF

### PRACTICAL MEDICINE;

BEING THE

### Spirit of the Public Journals,

FOREIGN AND DOMESTIC.

*Paucis libris immorari et innutriri oportet, si velis aliquid trahere, quod in animo fideliter hæreat.* SENECA.

Duo vitia vitanda sunt in cognitionis et scientiæ studio. ***** Alterum est vitium, quod quidam nimis magnam operam conferunt in res obscuras atque difficiles, easdemque non necessarias. CICERO.

1. *Hooping Cough—New Discovery.** Dr. Webster of Westminster, has discovered that the seat of hooping cough is in the *head.* He found, what many others found before, that the pectoral symptoms in this disease "are preceded and *accompanied* by pain of head, and fulness and redness about the temples and eyes." "I have thereby," says he, "been led to entertain the opinion, that the actual seat of the complaint may be in the head." His explanation of the ratio symptomatum is demonstration itself. The hooping *cough* is only to be considered "an effort of Nature to relieve herself by expanding the lungs to an unusual degree, thereby allowing a greater quantity of blood to flow into them, which may in some degree diminish the fulness and congestion now spoken of." It is odd that Nature has not pursued the same method (by hooping-cough) of relieving apoplexy, and the various other cerebral congestions to which we are liable. Perhaps this hint from Dr. Webster will induce her to follow his very ingenious mode of *emptying* the vessels of the head by reiterated paroxysms of *coughing !*

It is hardly necessary to remark, that Dr. Webster's therapeutics confirmed the truth of his pathology. Leeching the forehead and behind the ears did wonders—especially when assisted by open bowels—calomel and ipecacuanha—squills and antimony, &c.

We knew, long before Dr. Webster's doctrine appeared, that in children who die of hooping cough, there are generally found congestion and effusion in the head. But so far from supposing these to be the *cause* of the hooping cough, we deem them to be the *consequences* of obstructed circulation through the lungs, and of the determination kept up to the head by the successions of the hooping cough. But, although we look upon Dr. Webster's theory as highly fanciful, we by no means object to the practice, as it is calculated to

* Dr. Webster, Med. and Phys. Journal, December, 1822.

prevent injury in an important organ during the progress of hooping cough.

2. *Wounded Nerve.** In a former volume of the Medico-Chirurgical Transactions, Mr. Wardrop, who is a very able surgeon of this metropolis, related a case of severe symptoms arising from the prick of a thorn on the point of the finger, where amputation of the finger brought instant and permanent relief. The following case is considerably analogous.

"A young gentleman received a cut with a gun-flint obliquely across the radial side of the distal phalanx of the left thumb. The wound was about two thirds of an inch in length, and so deep as to divide the digital artery. Though accompanied with an unusual degree of pain, it readily healed by adhesion, and being considered of little importance, no further notice was taken of it. The patient, however, returning to his usual habits, and living rather fully, in a few days the thumb became painful, and the uneasy feelings, accompanied by constitutional irritation, had greatly increased when I first saw him, which was on the tenth day after the accident. No change could then be perceived in the appearance of the thumb, and the cicatrix seemed perfectly natural, notwithstanding he complained of great pain not only in the wounded thumb, but also in the fore finger and radial side of the middle finger, which extended up the arm, and as far as the neck and side. The pain was constant, and when the wounded thumb was even slightly touched, it became excessively severe. The pulse was frequent and tense, the face flushed, and the tongue white and frothy. A very copious general bleeding gave almost immediate relief; the pain in the arm and back decreasing, and the thumb becoming less painful to the touch. For three successive days all the local symptoms recurred, but yielded each time to a repetition of the bleeding, along with copious purging, and a strict antiphlogistic regimen." 206.

The patient continued to have paroxysms at times, which were occasionally excited evidently by mental emotions, or by taking small quantities of animal food. Opiates gave little relief, and nothing seemed essentially useful but strong purgatives and the most abstemious liquid food.

Three weeks having now elapsed from the date of the accident; the patient being considerably reduced in flesh and strength; the functions of the digestive organs being much deranged; the paroxysms of pain extending over the whole hand, arm, neck, and even down the back; it became imperative to try some bolder step: Mr. Wardrop therefore, in concurrence with Mr. Cline, made a complete division of the nerve above the injured part by means of a transverse incision close to the second joint. The operation was

---

* Case of Wounded Nerve of the Thumb followed by severe symptoms which were relieved by a division of the Nerve. By J. Wardrop, Esq. Surgeon Extraordinary to the King. Med. Chir. Trans. vol. xii.

instantly followed by a complete abatement of all the symptoms; but they were not permanently subdued for several weeks, during which time, whenever the patient took food of difficult digestion—when purgatives did not operate readily—or when the mind was by any means irritated, the pain attacked the hand and arm to a considerable degree. After this, however, his health became quite re-established, and more than two years have now elapsed since the operation. He can take great exercise in hunting and shooting; but what strongly points out the sympathy which an injured part sometimes preserves for a long time with the digestive organs is, that when from any cause the patient's stomach is disordered, he feels a pain in the injured thumb.

Mr. Wardrop's case is followed by some judicious pathological and physiological remarks on injuries of the nerves.

---

3. *Strictured Colon.** Mr. Sterry was called to a gentleman, (ætat. 44) on the 3d February, 1823, who complained of pain in the abdomen about the umbilical region, apparently of a colicky nature, the bowels being confined. Purgatives were given, but did not produce satisfactory effects. He was then bled, and had more aperients, which produced a few scanty motions. On the 10th, the uneasiness in the bowels and the constipation having increased, Dr. Walshman was called in, and more purgatives were prescribed, but without effect. In short, the united skill of Drs. Maton and Walshman, Sir Astley Cooper and others, was unable to give relief to the bowels, and the patient died in great misery after three weeks ineffectual medical treatment.

The following were the appearances on dissection.

"On opening the abdomen, all the intestines, excepting the rectum, were found exceedingly distended with air, insomuch that it was very difficult to cut through the peritonæum without doing injury to the intestines. Wherever the intestines were in contact with each other, red lines, from the adhesive inflammation, marked their disposition to unite. The derangement which produced death was situated at the upper part of the rectum; the sigmoid flexure immediately above it was much distended, and the cœcum enormously enlarged. At the termination of the sigmoid flexure of the colon, and at the beginning of the rectum, a stricture was found completely encircling and obstructing the intestine, which was very much ulcerated. This was the cause, and the *sole* cause, of the impediment to the passage of his motions, and of the fatal issue of the case.

"The inner coat of the intestine was also thickened, and had a carcinomatous appearance.

"No material quantity of the quicksilver was discovered, except in the small intestines, where a small portion was found adhering to

---

* Mr. Sterry, Med. Repos. No. 113.

the inner coat; the whole of the alimentary canal, however, was so loaded with a prodigious quantity of fermented matter, that it would have taken up more time than, under the particular circumstances of the case, was admissible, in order to arrive at any certain conclusion respecting the quantity of that substance actually contained in the different portions of the intestinal tube."

It seems very strange to us that a *stricture* should have produced such *sudden* effects, and that its existence should not have been indicated months and years before the patient's death. There is something very mysterious in the case.

---

4. *Memoire sur l'Œdème de la Glotte, ou Angine Laryngée Œdemateuse.* Par G. L. Bayle.*

It was our intention to have registered this interesting memoir long ago in our pages, but, till the present time, we have not been able to accomplish our wish. Perhaps it would be prudent, at all times, to direct our attention to the past as well as the present productions of the press—and not to allow the latter to wholly engross our pages. Facts are independent of dates—and therefore it must be desirable to occupy our limits with the best kind of matter, whether foreign or domestic—and whether of the current moment, or a few revolutions of the sun in arrear.

Some sudden and unexpected deaths, the symptoms of which were not alarming, naturally engaged our author to clear up the mystery by *post mortem* examination; and, in several instances, he recognized an occlusion of the larynx by different states of disease, as the cause of the melancholy event. Bonetus, Morgagni, Lieutaud, Vicq. de Azyr, and others, relate many instances of sudden death, determined by various lesions of the larynx; but among those lesions, in the experience of M. Bayle, that which was most frequent, most constantly mortal, and the least noticed, or described by authors, was this œdematous tumefaction of the borders of the glottis. It is a disease which he considers as necessarily mortal, if left to its course; but often remediable by art, if the means be early applied. Although it has never been well described, the description is very easy, because the symptoms are so characteristic and well-marked, that the diagnosis can hardly be mistaken.

He thinks the disease may be properly denominated "*angina laryngea œdematosa*," because it essentially consists in a serous infiltration of the membrane lining the larynx—the symptoms which it presents being the effect or natural consequence of the infiltration.

The complaint is characterized by a constant tightness or difficulty of breathing, from the œdema of the parts about the glottis, which œdema is not generally accompanied by pyrexia. *The inspiration*

---

* Nouveau Journal de Medicine, &c. Janvrier 1819.

*is difficult and sibilant, (sifflante,) while the expiration is easy*; and in its progress it occasions paroxysms of suffocation, during which the same difference exists between the inspiration and the expiration.

This œdematous laryngitis is a very different disease from that described by Boerhaave under the name of "angina aquosa," because this last affects the palate, tonsils, and pharynx, while the former has its seat in the larynx. The disease of Boerhaave is also far less formidable than the one under consideration.

There are some other diseases, however, which bear some resemblance, in their symptoms, to the *angina œdematosa*—namely, convulsive asthma, angina pectoris, acute laryngeal inflammation, and sometimes aneurism of the aorta. But the practitioner has only to compare accurately the symptoms in the definition with those presented in these last-mentioned diseases, and he will soon see the difference. Thus both in angina pectoris and the convulsive asthma of Millar, the sense of suffocation comes on suddenly, and is referred to the chest rather than to the larynx—neither does the difficulty of breathing continue in the intervals of those diseases. The *laryngitis œdematosa* is to be distinguished from *acute* inflammation of the larynx by the absence of fever in the former, and its presence in the latter. The march of the two diseases is also very different. The "*angina sicca*," mentioned by Areteus, Celsus, Sydenham, Boerhaave, and Van Swieten, is so vaguely described by those authors that it is difficult to say what it was. Boerhaave, however, asserts, that there is no sign of tumefaction, internal or external, either during life or on dissection. From this it would appear to be a spasmodic affection.

It is now proper to give some detail of the varieties of the angina œdematosa, its causes, progress, effects, and treatment, concluding with individual cases in illustration.

1. *Varieties.* This disease is either primary or consecutive, that is, unpreceeded or preceded by any other disease of the parts or neighbourhood. In both cases, however, the progress is the same; and when it is symptomatic, it may occasion death, though the primary affection be cured. In fact, in these complications the angina should be regarded as the principal affection, and it is against it that all our efforts should be directed. When idiopathic, the disease appears to be a catarrhal or inflammatory affection of the larynx—when symptomatic, it sometimes depends on an abscess in the larynx or neighbourhood, sometimes on an ulcer there—and sometimes it is a suite or sequence of other disease, acute or chronic, which determine œdema of the glottis by irritation of the parts.

2. *Causes.* The causes of *symptomatic* œdema anginosa are, of course, as numerous as the various maladies which produce it. In respect to the *idiopathic* species, it more frequently supervenes during convalescence from febrile diseases of a grave character, than under other circumstances. But whether it occurs in this way, or without any preceding indisposition, the *occasional or exciting causes* are not easily appreciated, or well understood. They are, in general, those which determine inflammatory and catarrhal affections, in individuals predisposed to such complaints. But in what this *predisposition* consists, it is not easy to say.

3. *Progress.* Angina laryngea œdematosa may commence with symptoms of suffocation, accompanied with pain in the region of the larynx; but usually its invasion is less alarming. It declares itself only by an uneasy sensation about the larynx, which the patients endeavour to free themselves of by strong and sonorous expirations to expel supposed mucosities about the throat. They frequently apply their hand to the part; or they complain of a tightness or uneasiness rather than pain there. The voice is somewhat hoarse—there is no fever—and, in other respects, the patient appears well. But in the course of two, three, or four days, the disease augments—the efforts, by hawking, to clear the throat, become more frequent—some glairy mucus is occasionally expelled—the voice gets hoarser, and is sometimes lost—there are momentary difficulties in breathing. Gradually the respiration becomes more noisy and shrill; yet the pulse remains unchanged—the appetite continues—and the patient considers his complaint a trifle. The next symptom, in order of succession, is cough, which is slight at first, and with considerable intervals; but after a few days (in some instances weeks) a new and more alarming symptom is superadded—namely, a sudden sense of suffocation, which lasts five or six minutes, sometimes a quarter of an hour—during which the inspiration is exceedingly difficult, the expiration easy. At the end of the paroxysm the breathing becomes somewhat freer, and an interval of some hours, or even days, succeeds. But the paroxysms recur, and with progressively increased violence, the respiration being less and less free in the intervals, and attended with more noise, especially when the patient is asleep.

At length the paroxysms of suffocation become violent, and life seems to be in imminent danger. The patients themselves are often affected with such anguish as to attempt their own lives, or request that their throats may be opened to afford them breath. The pulse now becomes irregular and intermittent. In one of these paroxysms, or, more commonly, in one of the intervals, the patient suddenly expires.

Angina œdematosa is almost always mortal. Our author saw seventeen cases of it in the course of six years, and only one of these recovered. The duration of the disease is very uncertain. Some of those, whose cases he noted, died from the third to the fifth day—others lived more than a month, and ultimately fell a victim to the disease, though the early paroxysms were slight, and the intervals considerable. In some instances the first paroxysm proved fatal.

4. *Post-mortem Appearances.* No sanguineous or serous engorgement or extravasation was observable in the brains of those who were examined. The edges (les bords) of the glottis were swelled, thickened, white, and infiltrated with a sero-gelatinous fluid, which could with difficulty be squeezed out by pressure of the fingers through incisions made by the knife. These swelled and infiltrated edges of the glottis were so disposed that every impulse of air from the mouth closed them, while impulses from the trachea opened them out again. Within the larynx only a slight and uniform œdema of the lining membrane was observable—sometimes a few red spots and injected vessels. Some organic alterations were occasionally observed about the chordæ vocales, the ventricles, or base of the cricoid cartilage. In some subjects there was an abscess in the larynx—in others, there has been found a carious state of the cartilages. The epiglottis is rarely natural—often very much swelled. The lungs are generally a little gorged, but crepitous.

Although the symptoms of angina œdematosa are not clearly described by authors, the effects of the disease are recorded in various writings. Morgagni and Bichat have given descriptions of states of the larynx evidently the result of this malady. It is hardly necessary to remark that death seems to result from lesion of the pulmonary function—that is, from non-oxygenation of the blood, which appears of a dark colour in the vessels of the corpse, and very little coagulated.

5. *Treatment.* Although, in every case where the symptoms were well marked, and the disease completely formed, death was the result; yet our author has observed, in other individuals, symptoms which led him to fear the existence of the malady in an incipient state, but which were dissipated by an active and revulsive treatment. Læennec has observed similar events. But as, in these fortunate cases, the disease was not fully formed and unequivocally characterized, our author will not be positive that they were really instances of the disease under consideration. He is anxious that the great danger of the disease should be always kept in view, and that the ultimate and most promising remedy (laryngotomy) should not be too long delayed, lest the function of the lungs be so far impaired that this last measure be rendered of no avail.

If the angina œdematosa be determined by, or complicated with, ulceration of the larynx or phthisis pulmonalis, we can only endeavour to soothe the symptoms, for the patient will succumb under the primary disease, even if the œdema could be removed. But in all other cases there may be a chance of recovery, provided the patient's life can be prolonged for a certain time.

The general means of treatment, then, are the following—viz. venesection, unless very strongly contra-indicated—leeches to the neck, anus, &c.—emetics, where the patient appears to have strength to support the measure—large sinapisms and blisters to the neck, arms, and nape of the neck, &c.—antispasmodics, and occasionally diuretics with diluents. "But as I know (says our author) that these means alone hardly ever succeed, when the disease is unequivocally characterized, and the suffocative paroxysms violent, so I think that laryngotomy should be practised, and that at an early period, lest the function of the lungs be irreparably damaged." But the question will naturally occur—what is this period? Our author answers that, so long as the paroxysms of suffocation are slight, and at considerable intervals, we should trust to the general means pointed out above; but that it may be laid down as a general rule, that tracheotomy is indispensable whenever one or more violent paroxysms of orthopnea have occurred in a patient whose voice is hoarse or extinct, the inspiration difficult, and expiration easy, with constant and notable tightness (gêne) in the breathing during waking and sleep. The urgency of the operation will be in proportion to the degree of this last symptom between the paroxysms, and to the shortness of the interval between the attacks of orthopnea.

6. *Cases.* Our author next proceeds to detail a few histories of individual cases, tending to shew that, however different were the states of the larynx giving origin to the œdema of the glottis, the symptoms preserved a surprising uniformity of character. He considers it sufficient to relate a small number of examples, on account of this uniformity, and he has only to observe further, that the majority of the cases which he witnessed of this disease, were witnessed also by several other physicians, among whom he cites Lænnec, Fizeau, Nysten, Martin, Clarion, Merat, Cayol, &c. We shall now endeavour to lay before our readers a portrait of these cases.

*Case* 1. A taylor, aged 25 years, of bilio-sanguine temperament, had been ill eight days when he was received into La Charite, on the 5th Brumaire. His complaint, he said, had commenced with suborbital cephalalgia, bitter taste of mouth, furred tongue, weight in epigastrio, frequency of pulse, and evening exacerbations. The disease pursued the same march after his entry into hospital, increasing, however, in force. In short, it turned out a "putrid bilious fever," as it was then termed, and by the 25th of the same month convalescence was completely established. On the 27th the patient was seized with dry cough, at considerable intervals—the voice was hoarse—and he felt a sense of tightness in the throat. 28th. He got up, but was constantly putting his hand to his throat—voice very hoarse and low. 29th. He had this day, at intervals, an extreme difficulty of breathing, at which times the inspiration was almost intercepted, and very noisy—the expiration free. The patient said he was choaking—his eyes were prominent and wild—his voice almost extinct. This state would continue for ten or twenty minutes, and then an interval of one or two hours of comparative freedom would usher in another attack. The night of the 30th was very distressing, and he was obliged to sit upright for hours together. 30th. The patient was out of bed, and at times appeared in tolerably good health; his appetite continuing, and there being no fever. But the difficulty of breathing progressively increased, and the pulse, which was regular and natural in the calm intervals, became concentrated, somewhat frequent, and at times intermittent, during the paroxysms of dyspnœa. The night of the 30th was very bad, and moments of furious despair were occasioned by the sense of impending suffocation. 1st. *Frimaire.* The patient up, as usual, and had some appetite —no fever—respiration exceedingly embarrassed and noisy—glairy expectoration, or rather expuition, from the larynx—little cough—

inspiration very difficult, expiration the reverse—the paroxysms of dyspnœa have shorter intervals, and are more violent in degree. The patient could be heard breathing at a considerable distance. The night of the 1st was less distressing than the preceding. 2d. The appetite still continued—no fever—thorax sounded well in all parts; but the patient was obliged to sit on his bed all the day. Two paroxysms only to-day. At nine in the evening he had a tremendous paroxysm, during which he cried out for a knife to cut his throat! At ten o'clock he appeared more calm, but presently expired. The body preserved its temperature for a long time.

*Dissection.* Brain sound. On opening the larynx from the pharynx the following appearances presented themselves. The epiglottis somewhat thickened, white, and rather infiltrated towards the edges. The orifice of the glottis somewhat contracted, but still presenting sufficient aperture for the admission of air. The edges or sides of the glottis were thickened, infiltrated, and gellatinous looking—the right edge formed a kind of loose hood or pad, which rose more than three lines higher than the opposite border, and formed a kind of valve, which completely closed the rima glottidis, when laid over it. The interior of the larynx was covered with a glairy mucus in great abundance. The chordæ vocales were swelled and infiltrated, and the ventricles of the larynx were almost effaced. In other respects all was sound and healthy in the trachea and chest, excepting that the lungs were rather tinged with blood, but perfectly crepitous. No mention is made of treatment in any part of the case; and we may fairly suppose that it was not of the most active nature, especially at that period.

*Case* 2. This was a man, 55 years of age, who had formerly enjoyed good health. On the 12th of July, 1808, and without any known cause, he was suddenly seized, at six o'clock in the morning, with affection of the throat, which threatened suffocation, and during which great noise was made in breathing. In all other respects the man was perfectly well. He had only been ordered some gargles, which had little or no effect. In the course of eight days the respiration became still more difficult—the tightness in the larynx was constant, and at intervals there was a great sense of suffocation, especially in the night. He entered La Charité on the 20th of the month, presenting the symptoms abovementioned—all the functions, excepting respiration, being healthy. From the 20th to the 30th of July the symptoms continued stationary; and leeches were twice applied *to the anus* during that time, *without any benefit.* He was ordered hydromel and juleps with much the same success. On the 1st, 6th, 11th, and 24th August, sinapisms were applied to the larynx. By these the symptoms were mitigated, and on the 24th August he had very little dyspnœa, but the vox rauca remained. He had not had any paroxysm for several days. In the beginning of September the patient found himself so much better that he wished to leave the hospital. He was kept till the 12th, to secure against relapse, and then was discharged.

Whether the above was actually a case of the formidable disease under consideration, must be left to the decision of the reader.

*Case* 3. Peter Salard, 28 years of age, of bilio-sanguine temperament, had a long illness, which was characterized as a putrid fever at La Charité. He was discharged however, as cured, and continued well for a few days, when he was seized with a new malady on the 4th of Thermidor. He first felt an uneasy sensation in the larynx, the voice becoming hoarse the same day. The inspiration was difficult, the expiration easy. To these symptoms was added a *cough,* though not very frequent—the symptoms themselves becoming more aggravated from day to day. His strength was gradually declining, and on the 10th Thermidor he re-entered the hospital La Charité. He now experienced great pains in the larynx—the inspirations were very difficult, the expirations easy—cough hard and frequent, accompanied with pain in the chest, which, however, sounded well in every part. The patient made frequent efforts to expel something from the throat, and some tenacious expectoration was discharged by these attempts. There was besides a considerable discharge of thin watery fluid from the mouth. The appetite was good, but deglutition was rather difficult. The fauces and tonsils were of a natural colour.

From the 11th till the 15th of the month, the symptoms went on increasing in severity, the emaciation making rapid progress. Yet his appetite was good, and he ate a considerable quantity of food daily, in spite of the difficulty of swallowing. In the evening of this day he went to bed, but was very unwell, and the dyspnœa increasing, he was obliged to get up; his breathing was so loud, that it could be heard all over the ward. About ten o'clock he was seized with a sense of suffocation, and suffered great anguish at times, which threw him into a state of despair and fury. At eleven o'clock these symptoms subsided, and in a few minutes more he expired.

*Dissection.* The pharynx was sound, and also the œsophagus. The right border of the glottis was found thickened, elongated, and infiltrated, so as to be capable of closing almost entirely the rima glottidis when drawn over the aperture. The lining membrane of the larynx was sound, presenting neither ulceration nor redness. The chordæ vocales were a little thickened and infiltrated. In the posterior parietes of the larynx, that is, between the mucous membrane and that of the pharynx, there was found an abscess, extending from the superior extremity of the arytænoid cartilages to below the middle of the cricoid cartilage. Part of the neighbouring cartilages were carious—the matter of the abscess was thick and white—the trachea and lungs were sound.

*Case* 4. This case is from the notes of the celebrated Lænnec, and the unfortunate subject was a student in medicine, of a good constitution, and who had previously enjoyed sound health. In the month of January, 1805, he was seized, after excessive fatigue, with a violent hæmoptysis, and some days afterwards with a putrid and malignant fever, which lasted 25 days. In the course of this disease he coughed a good deal, and complained of his throat. His convalescence, however, was rapid, and in a few days after the fever ceased he was able to get out of doors. His strength returned rapidly, his appetite was good. He ventured out on a wet day, and soon afterwards his voice became very hoarse, and he felt, at intervals, a difficulty of breathing, attended by noise in the act of inspiration, and some pain in the region of the larynx. Two or three days after this he experienced a severe suffocative paroxysm; and similar paroxysms returned the following days. In the intervals the patient found himself tolerably well, but his voice was extinct. He applied, of his own accord, a blister to the throat, which produced considerable relief. He had good appetite, but felt a choaking sensation, whenever any victuals passed into the œsophagus. About the sixth day from the commencement of the suffocative symptoms, all the phenomena became aggravated—inspiration was now constantly difficult and noisy—yet the appetite continued good. In the course of the following night he had two or three dreadful paroxysms which threatened his life. He sent for Beclard, Fizeau, and Lænnec, who found him in a deplorable condition. He had one or two attacks in their presence. About six in the morning the pulse began to sink and intermit, and the sense of suffocation became dreadful. Dr. Lænnec proposed tracheotomy, considering the disease as œdema of the glottis, of which he had seen several examples under M. Bayle at La Charité. The disease was supposed, from all circumstances, to be idiopathic, and therefore that the operation would afford the patient time for the original malady to subside. A distinguished surgeon was therefore hastily summoned, who agreed with the physicians that a mechanical obstruction to respiration existed at the top of the larynx, for which tracheotomy offered the best means of relief. He made an incision; but the patient cried out that the aperture was not large enough for his breath. The surgeon, then, determined to enlarge the wound upwards, and having introduced a director, he slit open the thyroid (he must first have gone through the cricoid) cartilage. But the suffocation continued nevertheless! The larynx was now examined by means of a sound, but only blood and frothy mucus issued from the orifice. Before the medical attendants could decide on any thing respecting this strange phenomenon, the patient expired, in seven minutes after the operation.

*Dissection.* The larynx and trachea having been carefully removed from the body, it was found that the first incision had fairly laid open the trachea two lines in extent. It was not so with the larynx. The cartilage had been divided, but not the mucous membrane lining the cavity of this organ. The borders of the glottis were œdematous, and almost entirely closed up the aperture when laid down. Each border presented several lobulated folds of unequal dimensions. The cellular tissue, external to the mucous membrane of the larynx, was also much infiltrated, and the mucous membrane itself participated, in several spots at least, in the same state. The infiltration was particularly remarkable in the ventricles of the larynx, and about the chordæ vocales, from each of which a small tumefied excrescence projected, that obstructed the entrance of air into the lungs, but offered little, if any, impediment to its egress. The inferior part of the larynx was free, excepting that its diameter was somewhat lessened by the turgid state of the lining membrane. In the posterior parietes of the larynx there was a small protuberance, which, when opened, discharged half an ounce of pus. The mucous membrane of the trachea and its ramifications was sound. The lungs were gorged with blood, particularly posteriorly; but otherwise they were crepitous and healthy. There was no other lesion in any part of the body.

There is something obscure, if not mysterious, about the foregoing case. It would appear that the director had passed up between the cartilages and the lining membrane of the larynx, and that when the sound was introduced afterwards, the same membrane receded before its point, and led the surgeon into an error in supposing he had freely laid open the cavity of the larynx. But what are we to say to the non-efficiency of the opening into the trachea? Læennec accounts for it by supposing that the patient's strength was too far exhausted for respiration through the artificial aperture. To this we cannot agree, and for a simple reason—the poor sufferer had strength enough to *cry out that the opening was not large enough.* "L'incision faite au lieu ordinaire, le malade cria d'une voix étouffée:—*que l'ouverture n'etait pas assez large.*" To us it appears evident, either that the opening was not large enough to admit air, or that the surgeon made the same mistake in the trachea as he did afterwards in the larynx—that he left the mucous membrane undivided. We grant, indeed, that the operation may be so long delayed as to render it inefficacious, however well performed; but, in the present instance, we do maintain that, had the aperture in the trachea been properly made and of sufficient width and length, the patient would have experienced, at least, a temporary relief by free access of air to the lungs.

*Case* 5. Pierre Bailly, a shoemaker, 45 years of age, of thin and bilious constitution, without any evidence of scrophulous disposition, and having never spat blood, stated that he had been affected with cough for the last five years, but had suffered from no other complaint except an ague, with which he had been harrassed in the months of September, October, and November, 1806. During this intermittent (a quotidian) the cough was aggravated, and attended with considerable expectoration of limpid mucus streaked with some white particles. He was at La Charité. The voice had become nearly lost, and complaint was made of pain in the throat, at each side of the thyroid cartilage. This pain was more felt during deglutition and coughing, at which time there was also a sense of uneasiness at the epigastrium. The breathing was become embarrassed—and even difficult, at certain times each day. On the 8th of November the patient experienced, for the first time, a paroxysm of dyspnœa threatening suffocation—and in a few days afterwards a repetition of the attack. On the 17th of November the fever of type ceased. On the 18th three paroxysms of dyspnœa, in one of which he fainted, and had some convulsive movements. On examination, the 20th of November, at La Charité, it was noted that the inspirations were long, distressing, and accompanied by a sibilant noise, great distortion of the features, and distention of the alæ nasi. The expiration, on the contrary, was quite free. The breath was fetid—the voice completely lost, and he could only speak in whispers. In the region of the larynx he experienced a sensation of tightness rather than pain; but, in the act of coughing, he suffered pain both in the larynx and epigastrium. The chest sounded well on percussion—the appetite was good, though the tongue was yellow—the stools were easy—the urine natural and abundant. From the 20th November till the 12th December, the symptoms above described continued; and to them was added a diarrhœa. The emaciation now made rapid strides. The patient complained frequently of a sense and fear of suffocation, especially in the night—the cough was very frequent and harrassing, and there was evidence of purulent matter in the expectoration. On the 12th December he expired.

*Dissection.* The aperture of the glottis was not much diminished, but the borders were infiltrated to four times their natural thickness, and when slightly pressed, completely closed the rima. The epiglottis was rather larger than natural. In the interior of the larynx, at the posterior portion of each ventricle, an ulcer was found—deep, and discoloured at bottom. That on the left side would contain a pea, and had destroyed a portion of the superior chorda vocalis, almost the whole base of the arytenoid cartilage, and a part of the cricoid. The ulcer in the right side took a similar direction, but not quite to the same extent. The lungs were tuberculated and infarcted—some of the tubercles being in a state of suppuration. In the large intestines were found two ulcerations of the mucous membrane, with raised and hardened edges, and their surfaces covered with pus. The head presented nothing remarkable.

*Case 6. Aneurism of the Aorta simulating Œdema of the Glottis.* Stephen Pillet, 48 years of age, tall stature and robust constitution, entered La Charité the 29th November, 1808, having been then, according to his own account, six months ill in consequence of suppression of the perspiration. He had cough, and expectoration of glairy mucus, his voice was obscured, and his breathing embarrassed and sonorous. At each inspiration, a kind of hissing noise was produced; the expiration, on the other hand, was free. The patient often felt an uneasy sensation about the larynx—the least exercise increased his dyspnœa, without augmenting, apparently, the action of the heart. The pulse was regular in the left arm, but no pulsation could be felt in the right. The digestive functions were natural, and no emaciation had taken place. For six months before his entrance into hospital, a seton had been established in the back of the neck, which diminished, he imagined, the dyspnœa. This seton had now been dried up. The only measures pursued at the hospital were diluent aperient drinks, and small doses of a sedative at night. Towards the middle of January, the cough and expectoration were sensibly diminished—the breathing freer and less noisy. The patient walked about the hospital all day, without augmenting the dyspnœa, and he ate with keen appetite. Yet emaciation crept on, though slowly; he had no fever, nor heat of skin—but always complained of tightness and uneasiness about the throat. On the 26th January, Pillet dined as usual, but was seized, soon after, with a violent paroxysm of orthopnœa, being unable to breathe excepting when leaning forward and resting on his arms. He made considerable noise at each inspiration—his face was suffused and purplish. A blister was applied to his throat, and towards midnight the paroxysm declined. Next morning he was in his usual state of health. Towards evening, another paroxysm of dyspnœa, still more violent than the preceding, came on, and did not subside as the former; he expired on the morning of the 28th January.

*Dissection.* On opening the larynx, to which part his painful sensations were always referred, no vestige of disease was to be seen. The pharynx and neighbouring parts were also in a state of perfect integrity. The lower portion of the trachea was compressed and flattened by an aneurismal tumour, larger than a man's fist, and rising from the root of the aorta. This tumour took a direction backwards, and did not touch the sternum or ribs. It thus compressed, and intimately adhered to, the trachea, occasioning all the phenomena before described, and ultimately death. Where the aneurism compressed the trachea, the cartilages of the latter were absorbed, and there was nothing but the mucous membrane to prevent the irruption of blood from the tumour. This would shortly have taken place, for the membrane was beginning to slough. Several specks of ossification were discernable on the inner surface of the aorta both in the thorax and abdomen. The origin of the right subclavian artery was drawn out of this course, in an oblique manner, nearly an inch, by the aneurism; and thus the jet of blood, at each ventricular contraction, was broken, so as to prevent the pulse being perceptible at the wrist. The artery, however, in its course along the arm, seemed undiminished in calibre, and the member itself was equally as well nourished as the corresponding one on the opposite side. The left lung, on whose bronchia the aneurism more especially pressed, was gorged with viscid mucus; the right lung was nearly natural.

We have now finished M. Bayle's Memoir, and hope it is not uninteresting or uninstructive. Some objection might be made to the term œdema, as conveying the idea of a passive infiltration, as in dropsical swellings of the ancles; whereas the œdema here treated of, is evidently a product of inflammation, resembling the gellatinous effusion which we sometimes see under a blister. The term œdema, however, is applied by Sauvages to various kinds of infiltrations, as milky, urinous, and even *purulent;* so that M. Bayle is justifiable in using the word as he has done. We consider the disease here described as nothing more than a subacute inflammation of the glottis or neighbouring parts, requiring the antiphlogistic treatment, and very active counter-irritation. We agree with M. Bayle, that tracheotomy ought to be performed in every case where life is threatened by suffocation, and where there is not evidence of disorganization of the larynx—for in this case there could be no hope of permanent benefit from the operation. Neither should tracheotomy be too long delayed; for the want of decarbonization or oxygenation of the blood may produce such lesion of function in the system as to render bronchotomy of no avail, if performed when the patient is in the jaws of death.

---

5. *Extirpation of Cancerous Uterus.** Dr. Sauter has published a case of total extirpation of a carcinomatous uterus, where the patient lived four months after the operation.

In October, 1821, Dr. Sauter was consulted by a poor woman, of Constance, aged 50 years, who had borne six children, and who had been, for several months, distressed with a constant menorrhagia. Dr. Sauter found at the cervix uteri a painful excrescence, which bled on the least touch. He tried various remedies without success, and in January, 1822, he proposed the operation of extirpation, to which she readily consented. She was taken to the hospital, and the operation was performed on the 28th of the above month. It was a horrible operation which lasted three quarters of an hour, and during which the bladder was penetrated, and the intestines brought in view! We will not detail the steps of this enterprise, for we do not think it will ever be imitated in this country. The poor woman bore the operation well, and did not loose above a pint and half of blood. The intestines being returned into the abdomen, and the vagina stuffed with charpee, the woman was put to bed, and did not suffer much pain during the first four days, but was troubled with vomitings, and the abdomen became swelled and tender. After this period the functions resumed their natural course, excepting that there was a discharge of urine from the bladder through the wound. On the 42d day the wound was entirely healed, and in three months and a half the woman left the hospital to rejoin her family. In seven days afterwards she returned to the hospital with cough, dropsy, and diarrhœa, of which she died, four months and some days after the operation. On examination, the bladder was found still open—the wound of the peritoneum, through which the intestines had descended, was quite whole, and no inflammation or adhesion in the abdomen or pelvis.—*Revue Medicale.*

---

6. *Painful Affection of the Brain.*† There can be no doubt that

---

* Annali Universali, 1823.

† Dr. Yeats. Journal of Science, April 1823.

the human frame, although subject to the laws of vitality in particular, is yet considerably under the influence of the general laws of inanimate matter. We are burnt by an intense sun, chilled by the easterly blast, relaxed and unnerved by damp weather, braced up by a dry and cold atmosphere. So again, the blood is not only circulated by an apparatus constructed on mechanical principles, but it is influenced in its circuit by the common laws of hydraulics afterwards. Who has not experienced the unpleasant gravitation of the fluids towards the head, when that part of the body is kept lower than usual? And who is not acquainted with the beneficial effects of cold applied to the head, and the erect posture in inflammatory and congestive affections of the brain? The case narrated by Dr. Yeats is elucidatory of this principle.

A gentleman, 40 years of age, complained of uneasy, languid sensations in the region of the stomach, with furred tongue, impaired appetite, uneasy digestion, frequent head-aches, irregular bowels, unhealthy alvine secretions. The pulse was not affected. He had lost flesh. By proper alteratives and aperients the digestive functions were improved; but the head continued to solicit attention, in consequence of the great uneasiness complained of there. Local detractions of blood frequently repeated, together with a constantly soluble state of the bowels failed to afford relief. The cerebral affection indeed had increased—the pulse became quicker and harder, and the patient described his sensations as follow:— "A pain, with heat, would commence in the back part of it, deep-seated, and would be diffused gradually throughout the whole of the back part of the brain, which would continue sometimes for two hours, causing insufferable distress within the skull, but confined, as it would seem, to the cerebellum, as the crown and fore-part of the head were not affected." A horizontal posture increased his sufferings, and was most acute about three or four o'clock in the morning, after sleeping a few hours. He also felt considerable giddiness and confusion in his head when he stooped upon any occasion. Sir Astley Cooper inserted a seton in his neck—he was confined entirely to vegetable diet—and he was desired to keep *continually* in the erect position, by sitting in a chair, and not going to bed at all. The head was shaved, and kept unremittingly wetted with a cold solution of muriate of ammonia in vinegar and water. This plan produced the most happy effects. At the end of a week he was so much better that the plan was gradually omitted, and he was able to sleep in the horizontal posture, and awake without pain. The seton was withdrawn at the end of two months. The only medicines he took during this time were supertartrate of potash and some purgative medicines.

---

7. *Cancer of the Lip.** In consequence of reading an account of

* Dr. Bull. Med. Repos. No. 114.

M. Richerand's operation for lip cancer, Dr. Bull, of Cork, was induced to pursue a similar measure in the case of a female, 60 years of age, who had an ulcerated cancer of the under lip of two years standing. It extended from one angle of the mouth to the other, and in depth nearly to the reflection of the mucous membrane from the gums to the lip, presenting a fungous-looking ulcerated surface, much everted, so as nearly to reach the lower margin of the chin when the jaws were closed. Dr. Bull operated in the manner directed by M. Richerand, (Periscope for September 1822,) excepting that he used a scalpel instead of flat scissors. He commenced by an external incision through the integuments, and extending beneath the tumour from one angle of the mouth to the other, in the form of a long crescent. In the next incision the whole of the diseased parts were removed at one stroke of the knife, cutting so low as the frenum of the lip. Lint was applied to the wound, when the arteries were secured; then a compress, and lastly a bandage. On removing the dressings on the third day, suppuration was found to be established; and, on the twelfth day, it was quite healed, not by granulation, "but by a process which, indeed, I did not expect, viz. eversion of the mucous membrane and approximation of it to the skin, with *considerable elevation* also, insomuch that there is hardly any appearance of cicatrization, and a new lip is actually formed, so that when the jaws are closed, the upper lip and the new surface approach each other so nearly, that the loss of parts appears quite inconsiderable." For the few first days the patient could not retain her saliva, "but she now possesses that power almost to its full extent."

Our readers will remember that another case corroborative of the above was lately laid before them in this Journal by Mr. Swan of Lincoln.

---

8. *Epilepsy.** Baron Larrey has published an interesting and instructive memoir on epilepsy, in the July number of our respected Parisian cotemporary the Revue Medicale, into the didactic part of which we are unable to enter, but shall merely abridge from it the following cases.

*Case* 1. Louis L——, 22 years of age, a grenadier of the ex-guard, entered the hospital in 1806, having two tumours on the head, in which there was fluctuation, and slight redness of the skin. He was in a soporose condition at the time of his entry, and had been subject to frequent attacks of epilepsy. The tumours being laid open, a caries was observed penetrating both tables of the skull, through which a slight pulsation was perceptible. The sufferings of the patient were somewhat mitigated, and the epileptic paroxysms were less frequent and less violent; but the caries made progress, and a fungous of the dura mater protruded, any degree of pressure

† Baron Larrey. Revue Medicale, Juillet 1822.

on which, would, at any time, reproduce epilepsy. The patient died about a month after his entrance into the hospital. On examination of the body, it was evident that he had had chancres; and Baron Larrey has no doubt of the disease being of a syphilitic character. He thinks, from experience of other cases, that the fatal catastrophe might have been prevented by a timely exhibition of anti-venereal remedies.*

*Case* 2. Barthelemi T———, 26 years of age, entered the hospital, for the treatment of two very large and scrophulous-looking tumours on the neck, accompanied by pains in the head, and paroxysms of epilepsy, to which last disease he had been subject, by all accounts, for some years. He stated, that in 1802 he had the venereal disease, and was treated methodically by a regular practitioner. Six years afterwards he was wounded in the thigh by a musket ball in Spain, and experienced both the hospital fever and hospital gangrene. As the wound in the thigh cicatrized, he began to be affected with violent pains in the head, which were relieved by a discharge of purulent matter from the ear. This discharge ceasing, he became deaf—had ringings in the ear—vertigo, and slight epileptic attacks. In this state he was sent to the hospital of the guard, where he was leeched, blistered, and given internal medicines, by which means the above-mentioned symptoms were dissipated. At this epoch an inflammatory swelling appeared in the arm-pit, which was (unfortunately, the Baron observes) discussed, instead of being brought to suppuration. From this time the epileptic paroxysms became extremely violent, and also frequent. The glands of the neck swelled, and some of them broke into abscesses. It was at this period that the patient came under the care of our author. Baron L. suspecting a syphilitic taint in the constitution, prescribed anti-venereal medicines, and applied caustic to the tumours. At the falling off of the eschars the patient experienced a tremendous attack of epilepsy, followed by hemiplegia of the *left* side, and an almost total abolition of his intellectual faculties. In a few days there was a renewal of the epileptic seizures, and the patient was in great danger. A large blister was ordered for the head. On shaving the scalp, a considerable prominence was perceived over the squamous portion of the right temporal bone, and another on the vertex. The blister produced some mitigation of the symptoms, and the patient was ordered diaphoretic drinks, with the use of the hydro-chloric acid, together with camphor and opium in large doses. The pains of the head were mitigated, and the paralysis sensibly diminished. A long perseverance in the acid, blisters, drains from the neck, and suitable regimen, brought about a complete recovery, the patient, however, experiencing many dreadful attacks of epilepsy during his progress towards

* A principal object of Baron Larrey, in this memoir, is to shew that a considerable number of epileptic affections arise from syphilitic and other vices of the skull.

a cure. The prominences on the scull entirely disappeared, and the patient was presented at one of the sittings of the Ecole de Medecine, with a cast of his head, such as it appeared on his first entry into hospital. Baron Larrey attributes the principal share of the cure to the internal use of the hydro-chloric acid.

*Case* 3. Francis P———, 24 years of age, entered the hospital on the 8th of October, 1821, for the cure of epilepsy, the paroxysms of which had become more and more frequent for some months past. Baron Larrey recognized, as the cause of this disease, a thickening (hypertrophy) of the cranium near the vertex, at which part there was a dull pain, especially on pressure. The other signs of cerebral apoplexy, in this case, were also striking, namely, debility of the moral faculties, languid countenance, drooping of the upper eyelids, inclination of the head forwards, stupid and taciturn manner. The epileptic attacks had been of long standing, eight or nine years, with intervals, however, of twelve or eighteen months. No blow, fall, wound, or other accident, could be traced as the cause of the disease, unless it was a retropulsion of scabies, which had been cured in three days by a shepherd of his native village. This epileptic being placed in one of the wards for wounded soldiers, was bled from the jugular vein, and had cupping glasses applied to the temples and sides of the cervical vertebræ. The head was shaved and blistered. Nevertheless he had an attack in a few days. The hydro-chloric acid was now prescribed in a ptisan, with taraxacum, calomel, and aloes, camphor, &c. New blisters and cupping were employed, and an issue was established at the nape of the neck. In the beginning of November two œdematous spots were observed on the crown of the head, to which the caustic potash was applied. On the 16th of November a fresh attack of epilepsy was experienced, but of a very mild kind. In other respects, the condition of the patient, moral and physical, is much ameliorated. In fine, by persevering for a long time in keeping drains successively from various parts of the head, the soldier was completely cured of his afflicting malady.

*Case* 4. This is a curious case in more than one point of view, as will be seen in the sequel. Vallet Baptiste, 35 years of age, a soldier of robust constitution, was admitted into the hospital of Gros Caillou on the 28th of August, 1821, with complete hemiplegia of the right side, caused by a fall which he had a month before on the left side of the head. Head-ache first resulted from this fall, and to such an extent as compelled him to go into the fever ward, where the usual depletions and derivations relieved the cephalalgia. Hemiplegia next succeeded, and for this he came into the surgical wards under our author. The right eye became affected, the tongue was paralysed, and the intellectual faculties were impaired.

After an attentive examination of Vallet's head, an unnatural protuberance was observed all over the left and superior portion of the cranium, and hence it was readily concluded by our author, that the phenomena presented by the patient resulted from the injury done to the cranium, meninges, and probably the brain itself, by the fall. It was therefore determined to attack the disease at its source. The vessels of the head were depleted by local bleedings—the scalp was shaved, and a blister applied all over the left side. In four days some relief was obtained even by these means. But successive blisters, issues, moxas, and various counter-irritants were perseveringly employed, besides a whole polypharmacy of internal remedies. The consequence was that, after one or two slight relapses, Vallet was discharged from the hospital towards the end of December, "*parfaitement gueri.*" Our author learnt, however, that this man had soon returned to the fever division of the hospital for febrile cephalalgia, and general bad health, where he died on the 7th February, 1822, seven months from his first coming under Baron Larrey's care for hemiplegia. The Baron had an opportunity of examining his body, and the following appearances presented themselves. The cranium sawed circularly, and carefully raised, shewed the left side of this dome much less capacious than the right. The surface of the dura mater, slightly injected, and of a yellowish tint, presented traces of chronic inflammation. The arachnoid was sound. The pia mater was highly injected, and manifestly in a state of inflammation. On raising the brain from the basis cranii, it was observed that the right optic nerve was larger, denser, and redder than the left—that a steatomatous tubercle, in a state of inflammation, encircled the basilary artery opposite the pons varolii, rendering the calibre of the artery at this place narrower than natural. The whole medullary substance of the cerebrum of the left hemisphere, was gorged with blood, and of a firmer consistence than that of the right, which, on the contrary, was nearly of a natural colour and consistence. The left side of the tuber annulare was also denser than the right. On examining the basis cranii they were all struck with the great difference between the two middle fossæ. The right was one half larger than the left.* In respect to the posterior fossæ, on which the lobes of the cerebellum rest, their shallowness excited the astonishment of all present. And this circumstance led our author to examine whether there was any correspondence between the genital organs and cerebellum. The lobes of this last did not appear to be more than one half the usual size of those in people of similar stature with Vallet. The testicles were not larger than a small bean each—and the penis did not admeasure more than six lines in length. This is a very strong fact for the phrenologists—and with this we shall close the present paper.

---

9. *(Retrospective) Case of Disease of the Colon, terminating in fatal Disorganization, mistaken, during Life, for Aneurism of the Heart.*† Dr. Duchateau, after some observations on the fallacy of diagnosis

---

* Baron Larrey sent this scull to his friend Soemmering.

† Duchateau. Bull. de la Societé Med. d'Em.

in organic affections, narrates an history, of which the following is a correct sketch; and which cannot be too strongly recommended to the notice of every practitioner.—A man, aged 25, of strong constitution and sanguineo-lymphatic temperament, subject to piles, and having quitted the army for a commercial situation in Paris, exhibited the following symptoms, when first visited by Duchateau :— countenance pale; lips discoloured; unnatural corpulence; chest and shoulders raised; respiration tight; and hoarseness. Exposed to great bodily exertion, he frequently came home, covered with perspiration, and breathless. He ate and drank well. Nothing was done. In March 1808, he was suddenly seized with suffocation, acute and dry cough, violent oppression, bilious vomitings, and syncope. Duchateau found him pale, apparently dying; pulse scarcely perceptible; tongue loaded with a yellow fur. Hence he suspected gastric disorder; but the man, on recovery, complained of intolerable palpitation of the heart, load at the stomach, constriction of the chest, and violent head-ache. The pulse was full, hard, irregular, particularly in the left arm; epigastrium tense; parieties of the chest elevated by frightful pulsations, and rendering an obscure sound on percussion. On being questioned respecting the palpitation, the patient said, that it first attacked him three years before, without evident cause; had been greatly aggravated of late; but that he had submitted to no treatment; *as his disease had been pronounced by all the physicians whom he had consulted, and particularly by Corvisart, to be aneurism of the heart.*

By the aid of medicine,* the man recovered his former state; but, aware of his situation, would submit to no restrictions. *Corvisart was again consulted, and confirmed his former diagnosis.* Another year passed without alteration, except that, for the last month, the oppression was aggravated; the cough more dry; palpitations more tumultuous; and walking impeded by a sense of painful anxiety about the præcordial region. On February 21st, 1809, after great exertion in cold weather, this man was seized with general uneasiness, convulsion, syncope, and expectoration of florid blood. Copious vomiting of red blood and black coagula ensued: and among the food, accompanying them, were observed some gland-like and fatty substances, compact, and of an ash-white colour, so as to resemble the pulmonary tubercle. They were washed, and preserved in vinegar.

Blood continued to be discharged both by vomiting and stool, and was always accompanied with portions of the substance just described. Convulsive spasm, slight delirium, unaccompanied by severe suffering, weakness and concentration of the pulse, extinction of the voice, insatiable thirst, and dryness of the tongue preceded death, which took place on the fourth day.

---

* The remedies prescribed by Duchateau, were successive applications of leeches to the throat, anus, and thorax; antispasmodics; purgatives, and laxative injections; chicken broth mixed with orange-flower water, and syrup of violets; and lemonade.

*Dissection on the 28th of February.* All the *thoracic organs were sound;* as were the stomach, liver, gall-bladder, spleen, and pancreas. On opening the intestinal tube from the pylorus, its duodenal and jejunal portions appeared healthy; but the ileum, for about two feet from its junction with the colon, was livid and gangrenous throughout. The colon itself was distended and blackish; its whole right portion sphacelated, and perforated to the extent of a crown-piece. The membranes were so much altered as to break beneath the finger. The whole adherent portion of omentum, which had escaped destruction, was of a dark-grey colour; the detached part had escaped into the intestine, and had followed the route of the fluids evacuated by stool and vomiting. The intestine, opened throughout, presented internally the same black colour; and the mucous membrane, the want of consistence, consequent on gangrene.

The *Reflections* of Dr. Duchateau we shall pass over, with merely noticing his opinions, that, in this case, part of the omentum had, at first, been the seat of an insidious chronic affection,* and that, contracting adhesions to the ascending colon, it had thus excited passive inflammation of the intestine, until the latter, perforated by the gangrene, had given passage to the portions of omentum, which were subsequently discharged by the mouth and rectum. Lastly, that the palpitations resulted from a lesion of some nervous branches of the mesenteric plexus sympathising with the nerves of the heart.

---

10. *Ulceration of the Cæcum, (private.)* A gentleman of good family attended the King on his tour to Scotland last year, and there received a strain apparently in the right groin, while jumping off a coach. Having also caught a severe cold at this time, he was laid up at Edinburgh with a large swelling in the right inguinal region, for which leeches, fomentations, aperients, &c. were used, and he got about again, so that he went shooting and using much exercise during last winter without any inconvenience. About the middle of May last a swelling appeared in the same place again, and an eminent surgeon of this metropolis was consulted, and considered it to

---

* We have, ourselves, no doubt but that the abdomen was, in this case, the seat of the primary disease, and that the physiological lesion of the heart and lungs was purely sympathetic. Asthma itself is frequently known to originate from epigastric derangement: and in persons of irritable heart, any irritation of the abdominal viscera, or cause accelerating the stream of blood in its transit through the venæ cavæ and lungs, will throw the heart into a state of violent and irregular action, very difficultly distinguishable from organic disease. Hence the practitioner cannot be too circumspect in delivering an opinion on such cases At this moment, there stands before our window, (in Warwickshire,) a healthy man, whom, many years ago, we gave up, as the victim of active dilatation of the heart, and who was subsequently cured, to our astonishment, by the aloes, spirit of ammonia, and bitters, of a sagacious empiric.—*Rev.*

be an abscess forming from which no danger was to be apprehended. It was opened on Sunday the first of June. On Monday he had a severe rigor, and on Tuesday much fever. On this day bark and other substances he had lately swallowed, were observed on the poultice, which alarmed the patient excessively; but the surgeon did not consider it as of serious consequence, for some days, though the fæces came constantly through the wound. Till Sunday the 8th of June, Mr. ——— thought the patient was going on well; but on that day he perceived symptoms which led him to apprehend more mischief than a mere communication with the intestinal canal. A physician was called in; but the patient rapidly sunk, and expired on Tuesday the 10th of June. The following were the minutes of dissection left with the family.

"*Appearances observed on inspecting the Body of S. S——, Esq. June* 11, 1823. In the lower part of the abdomen, immediately above the right groin, there was a cluster of enlarged absorbent glands connected with the abscess. The ulceration, which had taken place in the formation of the abscess had extended into the cæcum, or beginning of the great intestine, in consequence of which there was a large opening, forming a communication between the cæcum and the external wound.

"The inner membrane of the intestine in the neighbourhood of the ulceration, to a considerable extent, was in a state of inflammation, being of a very dark colour, in consequence of the vessels being loaded with blood. There were no other appearances of disease in the abdomen.

"In the chest, the lungs were found unusually turgid with blood; and the surface of the right lung towards the posterior part was covered by a thin layer of coagulated lymph. In the cavity of the pleura, on the right side of the chest, there were about six ounces of serous fluid."

☞ *Qy.* Was there not a hernia in the first instance?—*Rev.*

---

11. *Syphilitic Ulcers of the Larynx.** We remember hearing Mr. Hawkins' paper read and minutely discussed in the Medical Society of Windmill Street, where it excited considerable attention, on account of the talents of the young author, and the opportunities for observation which he enjoyed at the Lock Hospital of this metropolis. For these reasons we shall give a fuller analysis of the Essay than we are accustomed to give on similar occasions, as we wish to encourage merit, cherish zeal, and foster the spirit of observation in the rising generation of the profession.

That ulceration of the larynx occurs, and that not seldom, without any syphilitic taint in the constitution, is well known; but it is also proved by experience that venereal ulceration often attacks this

* Mr. Cæsar Hawkins. Med. and Phys. Journ. 290-1.

important part, though the disease is very little treated of by systematic writers on syphilis. Mr. Hawkins' observations are confined to those cases which occurred while house-surgeon in the Lock Hospital, and almost all the cases were accompanied by other venereal symptoms besides those of the throat.

The symptoms of ulceration of the larynx resemble, in a great measure, those of idiopathic inflammation—with this important difference, that, instead of arising in persons of robust health and inflammatory diathesis, they generally appear in persons of languid and unhealthy constitutions, or whose health has been broken down by the ravages of syphilis or the inordinate exhibition of its antidote. There are three kinds of ulcer in the throat, that are prone to affect the larynx. One is very rapid in its progress, and acute in its form —the second, or most common, often takes some weeks, or a still longer period before it arrives at any formidable height—the third, or least common, may be called the painful sloughing ulcer. In the first stages of these ulcers they are confined to the fauces—in the second stage they extend to the larynx—and if not fatal in one of these stages, they implicate the trachea or lungs, and terminate the sufferings of the patient.

"The first description of ulcer in the throat, which is apt to spread to the larynx, begins in the tonsils, in which a deep ulcer will be seen, containing a thick black slough, which so rapidly extends, that it deserves the name of the acute sloughing ulcer. The black sloughs do not separate and successively form, as is sometimes the case in the other species, but portions of the sound parts become every successive day involved in the slough, which hangs by shreds from the palate and pharynx; the secretion of saliva being very much increased. The countenance is, almost from the beginning, bloated, and of a dark venous hue, before the symptoms of ulcerated larynx begin, and consequently, before the blue colour can arise from any obstruction in the larynx itself. It rather appears to originate in the excessive languor of the circulation; a debility which it is impossible to remove, and which is such that the act of respiration itself appears to be too great a labour for the muscles to sustain. The slow passage of the blood through the lungs produces a copious secretion of mucus from the air-cells, which threatens to put an entire stop to the circulation. If the epiglottis and arytænoid cartilages become affected, the destruction of the patient is inevitable. The throat is so excessively irritable that not the slightest stimulus can be borne. The cells of the mucous membrane become gorged with serum, and fill up the aperture of the glottis; the parts having very much the appearance of an erysipelatous affection, to which disease this kind of ulceration really bears considerable resemblance, both in its progress and termination. Death may sometimes occur without the acute symptoms of ulcerated glottis, so that the patient may sink gradually till within the last few minutes.

"The second, or chronic ulcer, occurs chiefly in persons who have used mercury for this or some other form of the venereal disease; so that, perhaps, some doubts may be felt as to the origin of this species, even by those who grant the syphilitic nature of the acute kind. But, as few persons have suspicious symptoms of any severity without having taken mercury in some stage of their progress, except in situations where they are completely under the control of their medical attendants during the whole progress of the disease, the same doubts might be felt with regard to almost every other symptom of the venereal disease, particularly those usually called syphiloid or cachectic, if they had not each of them occasionally been observed after primary sores, for which no mercury had been exhibited. These observations particularly apply to the lower orders in the metropolis. It is not, however, of material importance to attach a name to the ulcer, as the employment of mercury is, in general, wholly inadmissible.

"This ulcer consists of a slough of a yellowish-brown colour, beginning generally in the pharynx, just behind the posterior arch of the palate, or else immediately behind the tonsil, on one or both sides. The slough is smooth and uniform in its appearance; it is not very deep, and the edges are on the same level with the surrounding soft parts. If it is on the increase, the immediate edge of the sound part has an irritable appearance, with a mixture of red and yellow streaks or spots, the red lines being gradually lost, so as to enlarge the size of the slough, without any apparent breach of continuity between the slough and the healthy pharynx. The local symptoms attending this ulcer in the pharynx are very frequently of an extremely mild nature, so that the whole pharynx may be covered with dark yellow sloughs, while the patient complains of little more than an uncomfortable sensation in swallowing, not actually amounting to pain; for, in fact, the ulceration itself is not the cause of pain, so much as the contact of the food with the ulcerated surface, which cannot take place till the healing process has begun, and the separation of the sloughs has commenced. But, in general, although the patient himself does not suffer very much, his system sympathizes with the extensive destruction which has ensued: the countenance is that of general ill health, with an exceedingly quick, weak, and irritable pulse, loss of appetite, broken unquiet sleep, and very great emaciation and debility.

"The last kind of ulcer which I shall describe is preceded, for two or three weeks before the appearance of any ulceration, by most excruciating pain, generally situated in the posterior part of the pharynx about the centre; the redness and other inflammatory appearances being very slight, but the difficulty in swallowing so very great that the patient's strength can scarcely be supported. At last a deep circular ulcer is perceived, which rapidly destroys the muscular part of the pharynx by sloughing, resembling the acute ulcer in appearance, and in being unaccompanied with any suppuration, but with more dryness of the mouth, and a fever, more of the inflammatory than of the typhoid character. In one case the acute pain was followed by the appearance of several little ulcers, which very quickly spread into each other. I have not had an opportunity of observing this last kind of ulcer, except in its first stage: I have been informed, however, on authority which I cannot doubt, that it is liable to extend to the larynx, though not so frequently as the other two which I have described." 276.

Allowing for some slight differences, the symptoms of the second stage of all these ulcers are so nearly alike that one description will suffice.

"With such symptoms as those which have been enumerated, the ulceration gradually proceeds till the epiglottis and arytænoid cartilages become involved in the disease. It is, however, somewhat remarkable, that the severity of the symptoms does not gradually increase in proportion to the extent of the ulcer, but continues of nearly the same degree of violence till the larynx is ulcerated; and then a fresh series of symptoms suddenly appear, constituting what I have called the second stage of the disease. For instance, a woman had extensive ulcers in the throat, which resisted every mode of treatment for many months. Nothing could rouse her constitution from the excessive languor into which it had sunk: but suddenly some accidental cause, such as a crumb of bread having fallen into the glottis, which had probably been some time diseased, caused a fresh activity in the ulcer, and gave rise to a new set of symptoms, which were fatal in a few days.

"At this period the voice loses its sonorousness, so that it becomes little more than a whisper, each attempt to converse producing a suffocating cough. Each act of inspiration is attended with a peculiar rattling noise, (different, however, from that of croup,) while that of expiration is comparatively easy. The muscles of the larynx and os hyoides are in constant and violent action, so that the larynx cannot be held steady for an instant; and at every inspiration the head is elevated, as if to give greater freedom to the entrance of the air, by straightening the tube through which it has to pass. If the difficulty of breathing be very great, the arms are tossed about in rapid and unceasing motion; while the eyes are fixed and projecting, or drawn up under the upper eye-lid, so as to hide the pupil. The sleep is sparing, and broken by starts, with the dread of impending suffocation. The countenance is generally pale, and expressive of the greatest anxiety; the face being covered with a greasy secretion, or bathed in profuse perspiration. The pulse is rapid in the extreme, so as scarcely to be counted; it is weak and excessively irritable; while the whole nervous system appears to be sunk in the depth of languor and debility.

"If the disease is accompanied with inflammation of the chest, the state of the pulse is liable to be affected. In one case, where there were repeated attacks of inflammation, the pulse, although weak in general, had the peculiar wiry feel of active inflammatory action; and in another, it was even full and hard. Such a combination of disease is particularly dangerous, as the treatment necessary for one may be improper for the other affection.

"But the most marked symptoms are those which affect the larynx itself. These are excessive pain and tenderness of the larynx, not general, but most commonly so far circumscribed that it is possible to distinguish whether the thyroid or cricoid cartilage is affected, and even which side of each individual cartilage: indeed, it is sometimes possible to cover with the point of the finger the spot which is the seat of the ulceration, as I have ascertained by dissection. When solid food is swallowed, it produces some degree of pain, but inferior to that of an acute inflammation of the pharynx, and in general is swallowed without material uneasiness; but the attempt to swallow fluids is invariably followed by acute pain and severe cough, arising, no doubt, from the insufficient closure of the glottis, in consequence of the morbid state of the muscles which regulate its actions; the irritation not being material from the deglutition of a solid morsel. Mr. Shaw, however, has recorded a case of ulcerated larynx, in which there was so much difficulty of swallowing that it was treated as a stricture of the œsophagus, till the employment of an opiate proved the difficulty to have been mere spasmodic. I have heard the difficulty of deglutition remarked as a distinction between ulceration of the larynx below the glottis, and an ulcer involving also the parts above the chordæ vocales,—that the difficulty exists in the latter case, but is not present in the former. That the difficulty of swallowing is greater where the pharynx is ulcerated, must be evident; and perhaps there may be none where the ulcer is below the glottis. The circumstance, however, to which I would attach importance, is not a difficulty of deglutition, but the fact of a few drops of fluid almost always entering and irritating the glottis; the rest of the liquid being swallowed without any effort. The fluid enters the glottis in cases where the epiglottis is entire, and persons have been known to swallow with tolerable ease after it has been destroyed; facts which might be expected from the experiments of Majendie, and which prove that it is the affection of the muscles which produce the irregular deglutition, and not the loss of the mechanical assistance of the epiglottis." 278.

These are the prominent symptoms—they are constant—but vary in intensity—their severity being by no means proportionate to the extent of the ulceration in different individuals.

Partial ossifications of the larynx sometimes take place so long after the preceding ulceration of the upper part of the throat, that it is doubtful whether it is an effect of the ulceration or a specific disease of itself.

"I have only happened to see two cases of this nature, though I have been informed of several others. In one, the greater part of the cricoid cartilage was changed into bone, some of which lay loose and carious in an ulcer of considerable extent. In the other, a man who died in St. George's Hospital, there was an abscess in the left side of the thyroid cartilage, very near the sacculus laryngis, containing several pieces of exfoliated bone." 278.

The symptoms, in these cases, are nearly the same as in common laryngeal ulceration, except the hoarse raucous voice. Between the symptoms, too, of ulcerated larynx and cynanche laryngea there is such considerable similarity, that some diagnostic characteristics become necessary. Although the ulcer may be out of sight, manual examination will sometimes discover it. If the finger is pressed firmly on the tongue, so as to avoid titillation, it may be passed round the epiglottis, and borne a sufficient time to ascertain the existence of ulceration, by the roughness or other deviation from the natural surface of the part. There are also some differences when the ulcer is below the rima glottidis. In cynanche, there is not the circumscribed tenderness of ulceration; there is much more pain in the deglutition of solid substances; and the swallowing fluids does not excite the peculiar action of the muscles of the glottis, or not in the same degree; there is also a difference in the voice.

"But one of the chief diagnostic marks between ulceration and simple inflammation of the larynx, is perceptible in the state of the circulation. The pulse in the latter, as in almost all acute inflammations, is full and hard; the countenance flushed, and the eyes suffused with blood; 'febris acuta et fere ardens, facies florida, deinde livescens.' The change of colour does not begin till quite the last stage of a very acute disorder rapidly coming to a crisis; the reverse of all these circumstances generally obtaining in ulceration of the glottis." 279.

Ulcers in the larynx vary in size from that of a split bean or almond to the diameter of an inch or more. The side of the arytænoid cartilage appears to be their principal seat—the epiglottis, however, is sometimes alone affected, and the largest ulcer Mr. H. has seen was situated in the posterior part of the cricoid cartilage and sides of the thyroid. The epiglottis is sometimes wasted, or even completely destroyed. The ulcer, in general, has a ragged irregular surface, the deeper parts being thickened so as, in some measure, to supply the loss of substance occasioned by the ulceration.

The third stage of laryngeal ulceration is where the lungs and trachea become affected. The difficulty of respiration produces such congestion in the lungs, that much watery and frothy fluid is poured out, and the blood is prevented from undergoing the proper changes. Sudden death, under these circumstances, is not uncommon. But by far the most common sequel of laryngeal ulceration is tubercular phthisis, which often terminates fatally in a very rapid manner.

Symptoms, however, of pulmonary consumption are occasionally produced by laryngeal ulceration, the lungs being perfectly sound. This has been denominated phthisis laryngea—the patient being worn out by long-continued cough and purulent expectoration. The ulceration, in such cases, sometimes extends down the trachea, and even into the bifurcations of the bronchia, the whole of the mucous membrane being irregularly studded with honeycomb-looking ulcers.

"It is asserted by some persons that it is most difficult, or altogether impossible, to distinguish between phthisis pulmonalis and phthisis laryngea. The cough, the purulent discharge, the emaciation, the hectic, are, it is true, common to both diseases; but still, I think, there are some marks by which they may be known. These are to be found in the peculiar form of the chest of consumptive persons; in the extent to which the patient has the power of expanding his lungs, which is almost always limited by the alteration of their structure, which takes place in phthisis pulmonalis; in the sound emitted from the chest by percussion, the change of tone being commonly perceptible by simple tapping with the fingers, even without the assistance of the stethoscope, to which so much importance is at present attached. Add to which the fact, that tapping the ribs gently will almost always excite coughing in persons whose lungs are unhealthy, while sound lungs will scarcely be affected by it." 362.

It is of the last importance to distinguish ulceration of the larynx from cynanche laryngea, between which diseases there is considerable similarity of symptoms, since the treatment of the latter will not avail in the former.

" The *acute sloughing ulcer*, which was first described, is of such a nature that it will often be fatal under the most judicious treatment. The depression of the whole system is so excessive, that the most powerful stimuli may fail to rouse the constitution; and, although they seem to be the only means to which we can have recourse, their exhibition requires perhaps considerable caution and judgment; for the inflammation of the throat itself is very high, while the general system is in the lowest state of debility; and there is so much local irritability, that the passage of medicine and food may of itself be sufficient to increase the rapidity of the sloughing.

" The best method, therefore, appears to be, to endeavour to soothe the parts, (for which purpose the steam from hemlock-leaves will often be found effectual,) while we support the system rather by nutritious diet than by strong cordials. Ammonia is of great service in such cases, and it may be given at the same time with sarsaparilla; which latter medicine is, perhaps, one of the most powerful means we possess for the support of the strength, independent of any antisyphilitic virtues which it may boast of. The best form in which we can exhibit it with this intention, is perhaps the syrup, which can be made of greater strength than the usual preparations, and acts more speedily upon the constitution; nor, perhaps, are the highly nutritious qualities of the sugar to be lost sight of. Its chief fault is a liability to offend the stomach; the tendency to which will, however, generally be counteracted by the addition of a little tincture of ginger, or some other aromatic. After the desired effect has been obtained, and the violence of the disease checked, some other form of the medicine, or some tonic, may be substituted for the syrup of sarsaparilla." 363.

In respect to the second species, the chronic sloughing ulcer of the throat, there are few diseases, so formidable in appearance, which are so completely under the control of medicine. As an illustration of this remark, our author mentions the case of a person who had an ulcer of this description, that had destroyed the tonsils, uvula, and part of the soft palate, occupying the whole back and sides of the pharynx, as high up the nares as could be seen. He had become greatly emaciated during the three preceding months, with an irritable pulse at 140, nocturnal sweats, and great prostration of strength. He took a pint of the Lisbon diet drink, and fumigated his throat twice a day with the red sulphuret of mercury. In ten days his pulse had sunk to 70, and had become quite soft and regular. His throat was nearly cicatrized, and he had already gained flesh considerably. No internal medicine, our author observes, can be employed with so much advantage as the sarsaparilla.

Our author has seen too little of the "*painful ulcer*" to recommend any thing with confidence from his own experience. Two cases of which he heard were cured by mercury, even although the disease had, in one of them, extended to the glottis. There are so few sloughing sores, however, with which mercury will agree, that the utmost caution is necessary in exhibiting it. Our author would rather be inclined to trust to large doses of opium, and at least delay the exhibition of the mercury till the excessive pain was relieved.

"Many persons have doubted the possibility of our effecting a cure, if the ulceration has once extended to the larynx itself. This opinion appears to me, however, to be erroneous, as, in one or two instances which I have examined, I have seen what had all the appearances of cicatrices from former ulcerations. This was particularly observable in the larynx of a woman who had all the usual symptoms of ulcerated larynx in a severe degree, and was discharged cured. She continued well for several months, and was then re-admitted into the Lock Hospital, with a return of the symptoms, accompanied with phthisis pulmonalis; of which she died. An ulcer was seen in one side of the glottis, with a ragged depression in another part of the larynx, which was probably the remains of the previous attack. Judging from their resemblance in feeling to some fatal cases, where dissection had verified the observation previously made with the finger, I believe that I have felt ulceration of the epiglottis and sides of the chordæ vocales, in persons who have been afterwards cured. In one very remarkable case, after the symptoms of ulcerated larynx had subsided, the cicatrix seemed to have so far contracted as nearly to close the glottis, producing very great difficulty of breathing, with a noise evidently showing the small size of the tube through which the air had to pass. The greatest relief was obtained by the occurrence of fresh ulceration in the lower part of the pharynx, which appeared to have in some measure re-opened the cicatrix of the former ulceration." 364.

There are two objects at which we ought to aim in the management of ulcerated glottis—the first, to relieve the excessive irritation—and next to heal the ulcer.

"Several plans may be tried in order to gain the first object; such as inhaling the vapour of a strong infusion of conium or opium, or of nitric acid diluted with warm water. If the ulcer can be touched with the finger, the solution of lunar caustic may be applied, in the manner recommended by Mr. Charles Bell, which appears to perform the same part here that it does in severe cases of spasmodic stricture of the urethra. It does not act as an escharotic, but simply diminishes the nervous irritability on which the contraction of the muscles depends. On the whole, however, I believe that fumigations of cinnabar are by far the most powerful means we possess of effecting this purpose. Very great relief is often experienced from the first time this plan is resorted to. Half-a-drachm or a drachm of the red sulphuret of mercury may be used once or twice a-day, according to the facility with which it is borne, or the benefit that is derived from it. The mercurial fumigation is not employed with a view to its specific action upon the system, for its effects are equally obvious when the constitution is not at all affected by it, as when the gums are made sore by its employment: salivation, indeed, to any great degree, would be disadvantageous, and perhaps dangerous. The action of the cinnabar rather appears to be local, though it would perhaps be difficult to explain exactly the manner in which it acts.

"It must be obvious that the use of the two latter remedies are chiefly applicable to those ulcers which follow the chronic ulcer of the throat; and that the soothing plan is most appropriate in the more acute and inflammatory species, while the violent local action exists." 365.

As a few hours are often of consequence, we may call in the aid of leeches and blisters to the throat—or even general bleeding, should circumstances require it. And having checked the most urgent symptoms, we are then to act on the ulcer by the sarsaparilla, or bark in large quantities, or ammonia, which is very useful in some cases.

We have now given a full account of Mr. Hawkins' valuable Essay, and we hope we shall often have occasion to notice productions from the same pen.

---

12. *Ancient Facts better than Ancient Fictions.* At page 222 of our last number we introduced a short *retrospective* notice of a practice pursued by Dr. Akenside, more than 60 years ago, in that Opprobrium Medicorum, cancer. On this notice the sagacious editor of the Medical Intelligencer, (June, 1823) makes the following reflection, with a note of admiration at the end. "What an extensive store of articles of intelligence would there accrue to any editor of a journal who might choose to fill up his pages from the various cases contained in the transactions of societies—*provided he were to hold novelty and importance of no value!*"

Now we appeal to the comparatively few who are in possession of the *fasti* of the profession, whether an "extensive store" of both valuable and important facts might not be collected from these sources, and which would be completely novel to ninety-nine in the hundred of medical society. But if the Medical Intelligencer means to insinuate, that this could be done with as little labour as swallowing down the mass of inanities and vanities which daily issue from the presses of London, Paris, and Vienna, we beg leave to differ from him. Nothing, in fact, but the labour and time necessary for such retrospections, prevents us from more frequently pursuing them. But now for "novelty and importance." In the very same number of the Intelligencer, which contains this sneer at bringing up a *fact* of 60 years standing, the editor of the Intelligencer, with singular inconsistency, tries back 200 years, and with much labour brings up a *fiction*. When Prosper Alpinus returned from his travels in the East, he entertained his friends in the West with some marvellous sights which he had witnessed on the road.—Among others, the exploits of a certain Arab Charlatan, named Haly, who played off some notable *slight-of-hand* tricks on the credulous of those days.* This Haly, in order to extract calculi from the bladder, without any cutting instrument, took a wooden cannula or tube, *eight inches in length, and one in diameter*, which he applied to the urethra, (quam colis canali admovebat) and blowing lustily (fortiterque insufflabat) puffed up the canal to the proper size, taking care, however, that the air should not enter the bladder, by pressing with his finger as far back as he could in the perineum—*consequently preventing any dilatation of the urethra between his finger and the bladder.* All that he had now to do was, to put a finger into the patient's rectum, and push the calculus quietly and easily through the neck of the bladder, and down along the urethra to that part which was previously dilated, when, of course, the extraction was performed without difficulty ! ! To any man, in the slightest degree acquainted with the anatomy of the parts, it would be an insult to say that the whole story is a fiction, being an anatomical impossibility. No man on earth could, by means of a finger in the rectum, push a calculus through the neck of the bladder, and down into the bulb of the urethra, where Haly's other finger was placed, to prevent air getting into the bladder. The whole was a juggling trick of Haly the Arab, whose impudence was only equalled by the credulity of Prosper Alpinus and the Medical Intelligencer.

When the subject of Sir Astley Cooper's forceps was under discussion at the Medico-Chirurgical Society, last winter, this passage of Prosper Alpinus was alluded to by one of the gentlemen present. The editor of this Review had the leaf containing the extract in his pocket, and it was only necessary to read the entire passage to the Society, to excite one universal burst of laughter at the Maunchausen story of Prosper Alpinus. Even the gentleman who quoted Alpinus did not attempt to defend him, but acknowledged that the operation was on a par with the cures of Prince Hoehenloe. So much for the " novelty and importance" of the *retrospections* in the Medical Intelligencer.

---

" * Eo tempore ' inquit' quo ego in Ægypto moram faciebam, Arabs quidam Haly vocatus ad extrahendos lapides sine incisione celeberrimus erat, quem ego sane cuidam duci Turcarum, Horam Bei vocato, multos lapides extraxisse vidi. Quo in opere absolvendo ille *ligneam cannulam* accipiebat, *longitudine octo digitorum*, et *latitudine digiti pollicis*. Quam colis canali admovebat, fortiterque insufflabat, atque ne flatus ad interiora perveniret, *altera manu extremum pudendi perstringebat*, foramen deinde cannulæ claudebat, ut virgam canalis intumesceret, et latior fieret, ac appareret · Quo facto minister digito in ano posito, lapidem paulatim ad canalem virgæ, atque in ejus extremum deducebat. Qui ubi præputio lapidem approquasse sentiebat, cannulam a virgæ canali fortiter impetioque amovebat, ut magna dexteritate lapis ad nuclei olivæ magnitudinem fuerit extractus : et ego interfui huic duci Turcarum, et postea duobus item Judæis, quorum alter puer erat, cui octo lapillos extraxit, et alter adultus, cui extraxit lapidem ad magnæ olivæ magnitudinem. Hic que est extrahendi lapidem e vesicâ modus, quo utebatur ille medicus Arabs."—Intelligencer, June, 1823.

